# Reproductive Outcomes of Dual Trigger versus hCG Alone in Women Undergoing In Vitro Fertilization with Fresh Embryo Transfer Cycles

**DOI:** 10.1155/2024/9972437

**Published:** 2024-07-09

**Authors:** Fatma Ceren Guner, Murat Ozekinci, Ibrahim Inanc Mendilcioglu, Zeliha Kasabali

**Affiliations:** ^1^ Department of Obstetrics and Gynecology Akdeniz University, Antalya 07058, Türkiye; ^2^ Department of Obstetrics and Gynecology Reproductive Endocrinology and Infertility Akdeniz University, Antalya 07058, Türkiye; ^3^ IVF Unit Department of Obstetrics and Gynecology Akdeniz University, Antalya 07058, Türkiye

## Abstract

**Aim:**

To investigate the effect of the triggering method on the results of fresh embryo transfer in patients who underwent gonadotropin-releasing hormone antagonist cycles.

**Methods:**

The study was conducted retrospectively at a university-based tertiary reproductive center. The sample consisted of a total of 295 patients, of whom 111 were in the human chorionic gonadotropin (hCG) trigger group and 184 were in the dual trigger group. The main outcome measure of this study was the live birth rate, and secondary outcomes were the implantation rate, clinical pregnancy rate, miscarriage rate, and good-quality embryo rate.

**Results:**

Patient demographics and baseline characteristics did not significantly differ between the dual and hCG trigger groups. The results also indicated statistically nonsignificant differences between the two groups in terms of the number of oocytes retrieved (*p* > 0.05), the number of mature oocytes (*p* > 0.05), and the fertilization rate (*p* > 0.05). The number of good-quality embryos (*p*=0.002) was higher in the dual trigger group compared with the hCG trigger group. However, the rates of clinical pregnancy and live births did not significantly differ between the groups (*p* > 0.05).

**Conclusions:**

Although the number of total and high-quality embryos obtained was higher in the dual trigger group, there were no significant differences between the two groups in terms of pregnancy outcomes. The fresh embryo transfer yielded similar rates of implantation and live births in both trigger groups.

## 1. Introduction

Oocyte maturation is the process by which the oocyte completes the first meiotic division and progresses to metaphase II. Oocytes, surrounded by granulosa cells, remain in the prophase of meiosis I until a surge of luteinizing hormone (LH) triggers meiosis to restart [1]. During the physiological menstrual cycle, surges occur in both LH and follicle-stimulating hormone (FSH). Gonadotropin-releasing hormone (GnRH) analogues (agonists and antagonists) are used to suppress pituitary function and prevent premature luteinization by inhibiting the production of endogenous FSH and LH during controlled ovarian stimulation.

Human chorionic gonadotropin (hCG), FSH, LH, and thyroid-stimulating hormone belong to the same glycoprotein family. Members of this group have the same *α*-subunit but different *β*-subunits. hCG, which has similar physiological activities to LH, is used during controlled ovarian stimulation to induce the luteinization of granulosa cells, final oocyte maturation, and resumption of meiosis [2].

The binding of the GnRH antagonist to the pituitary GnRH receptor is reversible. After GnRH agonist (GnRHa) injection, the receptor can be dissociated from GnRH antagonist and activated by GnRHa, allowing for the acute release of endogenous LH and FSH, which is known as the flare-up effect. The use of GnRHa alone compared to conventional hCG alone for final oocyte maturation in fresh embryo transfer cycles leads to severe luteal phase deficiency, and this has been associated with decreased pregnancy and live birth rates, as well as higher risks of early miscarriage, requiring considerable luteal phase support [3, 4]. However, the use of conventional hCG for final oocyte maturation does not lead to FSH surges [5]. The addition of GnRHa to hCG may stimulate both LH and FSH surges and improve oocyte quality and implantation through a more physiological effect. Recent findings suggest that normoresponders with a lower rate of mature oocytes have a significant increase in the number of oocytes and transferable embryos with the use of this dual trigger method. In addition, this method increases the proportion of oocytes obtained in relation to the number of preovulatory follicles. However, the effect of this method on pregnancy rates remains inconclusive [6, 7].

This study aimed to compare the outcomes of ovarian stimulation and the rates of clinical pregnancy and live births between hCG trigger alone and dual trigger procedures in a general population undergoing GnRH antagonist in vitro fertilisation/intracytoplasmic sperm injection (IVF/ICSI) treatment with fresh embryo transfer cycles.

## 2. Materials and Methods

The study was conducted at a university-based tertiary reproductive center. A retrospective database review was performed at the Department of Assisted Reproduction of the Akdeniz University Faculty of Medicine. All the GnRH antagonist IVF cycles performed on individuals aged 21–41 years from December 2017 to May 2018 were reviewed for eligibility for inclusion in the study. The patients who received treatment due to severe male factor infertility, those with malignant diseases, and those with <4 antral follicles on the third to fifth days of the menstrual cycle were excluded. After applying these criteria, a total of 295 patients were included in the sample. Using a two-sided significance level of 0.05, we determined that a sample size of 79 subjects in each group would afford 90% power to detect a significant difference in clinical pregnancy rates. This calculation was based on a control group proportion of 0.24 and a study group proportion of 0.46 [8]. The study was approved by the Institutional Review Board of Akdeniz University (2012-KAEK-20/437). The requirement for patient consent was waived due to the retrospective nature of the study and the absence of any interventions.

The starting gonadotropin dose was based on patient age, body mass index (BMI), antral follicle count, day 3 FSH level, and previous response to gonadotropins. Patient response was monitored by serial transvaginal ultrasounds for follicular measurements and serum estradiol (E2) levels during the IVF cycle. The GnRH antagonist cetrorelix (Cetrotide, Merck Serono, Germany) was started at a daily dose of 0.25 mg subcutaneously once a follicle reached ≥12 mm in diameter or serum E2 was >300 pg/ml and continued until the day of oocyte maturation trigger. The choice of either the HCG trigger or dual trigger was based on the physician's preference. During the trigger procedure, the patients were administered either 500 *μ*g hCG (Ovitrelle, Merck Serono, Italy) or 250 *μ*g hCG and 0.2 mg triptorelin (Gonapeptyl, Ferring, Germany) subcutaneously. Transvaginal ultrasound-guided oocyte retrieval was performed 35-36 hours after the trigger injection. The embryo classification was made based on the 2011 ESHRE Istanbul Consensus [9]. All embryo transfers were undertaken under transabdominal ultrasonography guidance. Luteal phase support was initiated on the day after oocyte retrieval and continued until a negative pregnancy test or 10 weeks of gestation.

The rate of implantation was defined as the ratio of the total number of transferred embryos to the total number of gestational sacs visualized. Clinical pregnancy was defined as the number of fetal heartbeats observed within six to seven weeks of pregnancy. The rate of live births was calculated as the ratio of the number of live births to the total number of patients who underwent embryo transfer.

The Statistical Package for the Social Sciences (IBM SPSS) version 23.0 was used for the statistical analysis of the data obtained from the study. To describe the sample, continuous variables, such as age, BMI, FSH, E2, and gonadotropin dose, were expressed as the mean ± standard deviation and median (minimum-maximum) values, while categorical variables were expressed as numbers and percentages. The normality of the distribution of continuous variables was analyzed with the Shapiro–Wilk test. Since parametric test assumptions were not met for age, BMI, FSH, E2, and gonadotropin dose, the differences between the means of two independent groups were analyzed using the Mann–Whitney *U* test. The differences in the percentages of categorical variables were analyzed with the Pearson chi-square test, and Fisher's exact test was used if more than 20% of the expected frequencies had a value of less than 5. A margin of error (*α*) value of 0.05 (or 95% significance level) was used to determine the differences in these analyses.

## 3. Results

**Figure 1:**
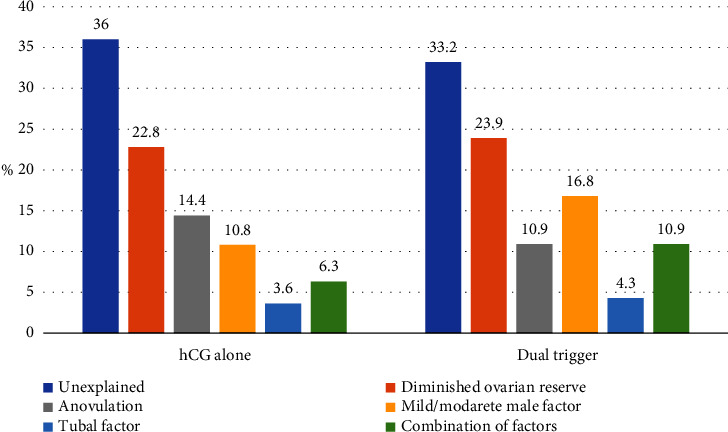
Etiology of infertility.

The study included a total of 295 patients, of whom 111 were in the hCG trigger group and 184 were in the dual trigger group. Patient demographics and baseline characteristics are presented in Table 1. There were no statistically significant differences in the baseline characteristics of the two groups (Table 1). In addition, no significant difference was observed in the distribution of IVF indications.  Figure 1 shows the etiologies of infertility in both groups.

Cycle parameters and embryological data are shown in Table 2. Although the dual trigger group had a higher number of oocytes retrieved (9.7 ± 5.6 vs. 11.6 ± 7.8; *p* > 0.05) and a higher number of MII oocytes (6.5 ± 4.5 vs. 7.6 ± 5.3; *p* >  .05), the difference was not statistically significant. Neither group had any superiority over each other in relation to the number of two distinct pronuclei (2PN) embryos or fertilization rates (Table 2). However, the total number of obtained embryos was notably higher in the dual trigger group than in the hCG trigger group (3.5 ± 3.1 vs. 3.9 ± 3.2; *p*=0.041). Analysis of embryo quality between the two groups revealed a significant increase in the number of good-quality embryos in the dual trigger group compared to the hCG trigger group (*p*=0.002). Moreover, the dual trigger group exhibited a significantly higher number of cryopreserved embryos compared to the hCG trigger group (*p*=0.012).

Fresh embryo transfers were performed in 74 patients in the hCG trigger group and 122 patients in the dual trigger group. A total of 90 fresh embryos were transferred to the hCG trigger group at an average rate of 1.22 embryos per transfer, while the dual trigger group received 159 embryos with an average of 1.30 embryos per transfer, showing no statistically significant difference between the groups. 21 gestational sacs in the hCG trigger group and 36 gestational sacs in the dual trigger group were detected by transvaginal ultrasound. Three patients in the hCG trigger group and four patients in the dual trigger group had miscarriages before reaching the 20^th^ gestational week. Among the patients who underwent hCG trigger, 18 women reached ≥24 weeks of gestation, and all had a live birth. Although 30 patients reached ≥24 weeks of gestation in the dual trigger group, two patients (one at 25 weeks and the other at 31 weeks) had stillbirth.  Table 3 shows the IVF and gestational outcomes of the groups. The hCG and dual trigger groups had similar results in terms of the rates of implantation (23.33% vs. 22.64%; *p* > 0.05), clinical pregnancy (28.38% vs. 27.87%; *p* > 0.05), miscarriage (14.29% vs. 11.76%; *p* > 0.05), and live births (24.32% vs. 22.95%; *p* > 0.05).

## 4. Discussion

In this study, we investigated whether combining GnRHa with standard hCG for final oocyte maturation could improve ovarian stimulation outcomes and clinical pregnancy and live birth rates in fresh embryo transfers performed on a general IVF population in GnRH-antagonist IVF-ICSI cycles. The study included a total of 295 patients who were divided into two groups as follows: those who received GnRHa at the final oocyte maturation (dual trigger) and those who only received the hCG trigger. There was no significant difference between the two groups in terms of age, BMI, basal FSH, LH, antral follicle count, duration of infertility, or comorbidities. Stimulation duration and total gonadotropin requirements were also similar between the groups. However, according to the results of the study, the total number of oocytes obtained and embryo quality were statistically significantly higher in the dual trigger. On the other hand, there were no significant differences in the rates of mature oocytes, fertilization, clinical pregnancy, or live births between the two groups.

The use of hCG alone in controlled ovarian stimulation shows its effect through its long half-life LH activity [10]. Unlike hCG, the use of GnRHa to induce ovulation stimulates the endogenous release of LH and FSH from the pituitary gland, partially mimicking the natural menstrual cycle. Although the effects of FSH increase in midcycle have not been fully elucidated, one of its important functions is to optimize corpus luteum function by increasing LH receptors in granulosa cells [11]. Studies have also shown that FSH is involved in nuclear maturation and cumulus-oocyte complex expansion [12, 13]. To investigate the positive effect of FSH on the results, Lamb et al. used bolus FSH together with hCG and found that the fertilization rate was higher in patients receiving FSH [14]. Poor oocyte quality or fewer mature oocytes are associated with unsuccessful results in in vitro fertilization. It has been suggested that the use of GnRHa increases the number and rate of mature oocytes obtained by creating an environment that is more similar to natural physiology. In a retrospective study including 27 patients, Griffin et al. applied the dual trigger method with the antagonist treatment protocol in the next cycle in cases where the hCG trigger had been applied in previous stimulation and reported the retrieval of more than 25% of immature and germinal vesicle oocytes [15]. The authors showed that the addition of GnRHa to hCG significantly increased the number and rate of mature oocytes. Similarly, a retrospective study including patients of similar characteristics was conducted by Fabris et al., who evaluated the addition of GNRHa to HCG and examined 81 patients [16]. They detected an increase in the number and ratio of metaphase II (MII) oocytes with the use of the dual trigger method. Although Li et al. reported that the dual trigger method positively affected the number of oocytes collected from patients with high ovarian response, they did not provide information about the number and ratio of MII oocytes [17]. In another study conducted with normoresponders, the number of 2PN embryos and good-quality embryos was found to increase with the dual trigger method, while no significant difference was observed in relation to the total oocyte count or the count and ratio of MII oocytes [18]. Seval et al. reported a statistically significant difference in the number of oocytes and MII in the dual trigger group [5]. In a randomized controlled study, Kim et al. found no significant difference between the groups in terms of the number of oocytes obtained [19]. Similarly, randomized controlled studies conducted by Mahajan et al. with 76 patients and Decleer et al. with 120 patients reported no significant difference in the number of MII oocytes [20, 21].

Zhou et al. showed an increase in the number of 2PNs, but a meta-analysis found no correlation between the addition of GnRHa and the number of 2PNs [18, 22]. Three retrospective studies comparing the fertilization rate found no positive effect of the dual trigger [15–17]. In our study, no superiority was found between the two groups in terms of the number of MII oocytes, the number of 2PN embryos, or the fertilization rate.

In the literature, there are studies that have compared the effects of dual and hCG trigger methods on embryo quality and, consistent with our study, have shown that the number of good-quality embryos is higher in the dual trigger group [5, 17, 21]. However, there are also reports indicating that this method has no superiority to hCG alone [19, 23].

Several studies have shown that GnRH receptors are found not only in the pituitary gland but also in extra pituitary areas, such as the endometrium, myometrium, fallopian tube, ovary, placenta, and unimplanted embryos [24–27]. GnRH modulates matrix metalloproteinases in placental trophoblasts. Matrix metalloproteinases are involved in extracellular matrix degradation and trophoblast cell invasion [28]. It has been shown that HOXA-10 expression, which acts as a modulator of endometrial receptivity in endometrial stromal cells, significantly decreases in patients who have undergone antagonist cycles compared to those who have undergone natural or agonist cycles [29]. Devroey et al. attributed the low implantation rate in the antagonist cycle to these changes in endometrial receptivity [30]. Another study supporting this information belongs to Bukulmez et al., who compared agonist and antagonist cycles and observed a low clinical pregnancy rate in patients treated with GnRH antagonist therapy although embryo quality did not differ [31].

Schachter et al. suggested that GnRHa had a higher receptor affinity than the antagonist [32]. They hypothesized that implantation might be improved through the postreceptor effect in endometrial cells as a result of the binding of GnRHa to the receptor blocked by the antagonist due to its high receptor affinity. In the same study, the authors found the pregnancy and ongoing pregnancy rates to be significantly higher in the dual trigger group, but they did not include live births in their study [32]. In a retrospective analysis of 378 patients who underwent embryo transfer, Lin et al. found the rates of implantation, clinical pregnancy, and live births to be higher in dual trigger procedures but did not observe any difference in miscarriage rates [23]. Similar results were shown by Kim et al. in a randomized controlled study evaluating 120 patients [19]. In another study, the rates of clinical pregnancy and implantation were found to be higher in those who underwent dual trigger [33]. Decleer et al. [21] reported that the implantation and ongoing pregnancy rates were similar between the groups, as in our study.

The primary limitation of this study lies in its retrospective design. Furthermore, the choice of trigger for each patient was at the discretion of the treating physician. Cumulative live birth rates were not incorporated into our article. Had we examined cumulative live birth rates, a notable difference might have emerged, given the higher number of high-quality embryos and cryopreserved embryos in the dual trigger group. Other constraints of the study encompass its singular center design and a relatively modest participant size.

## 5. Conclusion

Our investigation revealed a statistically significant enhancement in the percentage of high-quality embryos obtained with the implementation of the dual trigger protocol. Despite this observed improvement, no significant disparity emerged between the two groups concerning pregnancy outcomes. Notably, the rates of implantation and live births following fresh embryo transfer demonstrated similarity in both trigger groups. These findings suggest that while the dual trigger protocol positively influences embryonic quality, its impact on overall pregnancy outcomes, particularly in terms of implantation and live birth rates, appears comparable to the hCG-alone trigger. Further nuanced analyses and larger-scale studies may provide deeper insights into the clinical implications of our observed improvements in embryonic quality.

## Figures and Tables

**Table 1:** Patient demographics and baseline characteristics.

	hCG alone (*n* = 111)	Dual trigger (*n* = 184)	*P* value
Age (year)	32.5 ± 4.7	32.1 ± 4.6	0.431
Body mass index (kg/m^2^)	25.7 ± 4.9	25.8 ± 5.3	0.933
Baseline serum FSH (mIU/mL)	8.3 ± 3.3	8.0 ± 3.4	0.252
Baseline serum E2 (pg/mL)	48.6 ± 39.4	49.6 ± 35.3	0.800
Basal follicle number	13.4 ± 8.7	15.2 ± 11.6	0.412
Infertility causes including PCOS (%)	4 (3.6%)	7 (3.8%)	>0.999

E2, estradiol; FSH, follicle-stimulating hormone; hCG, human chorionic gonadotropin; PCOS, polycystic ovary syndrome. *Note*. Values are expressed as the mean ± standard deviation and percentage.

**Table 2:** Cycle parameters and embryological data.

	hCG alone (*n* = 111)	Dual trigger (*n* = 184)	*P* value
Total dose of gonadotropins (IU)	3338.9 ± 1335.9	3105.2 ± 1265.4	0.193
Total stimulation duration (day)	10.4 ± 2.0	10.2 ± 1.8	0.325
E2 on trigger day (pg/mL)	1981.9 ± 1077.8	2222.4 ± 1186.7	0.140
Number of oocytes retrieved	9.7 ± 5.6	11.6 ± 7.8	0.085
Number of MII oocytes retrieved	6.5 ± 4.5	7.6 ± 5.3	0.092
The rate of mature oocyte	65.7 ± 23.5	66.9 ± 25.4	0.746
Number of 2PN embryos	3.5 ± 3.0	3.9 ± 3.2	0.352
Fertilization rate, %	53.8 ± 29.9	52.7 ± 28.6	0.792
Number of embryos obtained	3.5 ± 3.1	3.9 ± 3.2	**0.041**
Good-quality embryos, % (*n*)	69.8 (88/126)	83.7 (226/270)	**0.002**
Cycles with fresh embryo transfer, *n* (%)	74 (66.67%)	122 (66.30%)	0.949
Number of embryos transferred per patient	1.22 ± 0.41	1.30 ± 0.46	0.185
Number of embryos cryopreserved	0.28 ± 0.07	0.61 ± 0.09	**0.012**

E2, estradiol; hCG, human chorionic gonadotropin; MII, metaphase II; 2PN, two distinct pronuclei. *Note.* Values are expressed as the mean ± standard deviation and percentage. *P* values in bold are statistically significant.

**Table 3:** Outcomes of IVF cycles and pregnancy.

	hCG alone	Dual trigger	*P* value
Implantation, % (*n*)	23.33 (21/90)	22.64 (36/159)	0.901
Clinical pregnancy, % (*n*)	28.38 (21/74)	27.87 (34/122)	0.936
Pregnancy at >24 weeks, % (*n*)	24.32 (18/74)	24.59 (30/122)	0.967
Miscarriage, % (*n*)	14.29 (3/21)	11.76 (4/34)	>0.999
Live birth, % (*n*)	24.32 (18/74)	22.95 (28/122)	0.826
Preterm delivery, % (*n*)	22.2 (4/18)	10.71 (3/28)	0.407

## Data Availability

The data used to support the findings of this study are available from the corresponding author upon reasonable request.
